# Clinical characteristics and differential diagnosis model of IgAV with gastrointestinal symptoms and acute abdominal disease: an observational controlled study

**DOI:** 10.3389/fimmu.2026.1899915

**Published:** 2026-08-28

**Authors:** Yu Zhang, Yang Liu, Zhiling Li, Wenzhong Niu, Zhixin Zheng, Yuhan Gu

**Affiliations:** 1 Department of Clinical pharmacy, Nanyang Central Hospital, Nanyang, China; 2 Key Laboratory of Clinical Pharmacy and Drug Metabolism, Nanyang, China; 3 Department of Pediatric Neurology, Nanyang Central Hospital, Nanyang, China; 4 Department of Clinical Pharmacy, Shanghai Children’s Medical Center, Shanghai Jiao Tong University School of Medicine, Shanghai, China; 5 Department of Pediatric Nephrology and Endocrinology, Nanyang Central Hospital, Nanyang, China

**Keywords:** diagnostic study, differential diagnosis, gastrointestinal symptoms, IgA vasculitis, neutrophil-to-lymphocyte ratio

## Abstract

**Background:**

Gastrointestinal symptoms of IgA vasculitis (IgAV) patients are relatively common. These symptoms are similar to those of acute abdomen, coupled with the poor expressive ability of young children and children, which leads to the possibility of misdiagnosis and delayed diagnosis.

**Methods:**

This is a retrospective observational controlled study. All the patients were treated in Nanyang Central Hospital. The demographic, medical history and laboratory tests data were collected from medical records. The characteristics of patients with IgAV have been described. Based on logistic regression, the diagnostic features of IgAV with abdominal symptoms were identified, and a diagnostic model was established.

**Results:**

IgAV patients with abdominal symptoms usually have an onset age of over 4 years old and are more common in autumn and winter. Compared with patients with acute abdomen, patients with IgAV have lower indicators related to inflammation and a lower proportion of fever, nausea or vomiting, abdominal distension, and changes in borborygmi. Based on these characteristics, 10 related diagnostic features were identified, and a robust diagnostic model was also established, which may be helpful for clinical diagnosis of IgAV patients.

**Conclusions:**

The advantage of this study lies in the establishment of a model and nomogram by integrating clinical symptoms and laboratory test data, which has good medical interpretability and clinical practicability.

## 1 Introduction

IgA vasculitis (IgAV) is caused by the deposition of IgA immune complexes in the small blood vessels, and it often leads to purpura on the affected skin. Apart from the skin, the small blood vessels in other organs are also affected (1). Therefore, patients with IgAV will also develop arthritis, gastrointestinal symptoms and kidney damage (2). Gastrointestinal symptoms are relatively common, but their incidence varies among different studies (3). The lesion sites of patients with gastrointestinal symptoms are mainly concentrated in the duodenum, presenting as red mucosa, bleeding, erosion and even necrosis (2). Therefore, these patients often present with symptoms such as vomiting, abdominal pain, and gastrointestinal bleeding. Vomiting and abdominal pain have been reported in about 65% of IgAV patients, and gastrointestinal bleeding has been reported in 30% of patients (4). About 20% of the patients with serious and life-threatening symptoms (5).

Although there are still no good diagnostic markers at present, the diagnosis of IgAV is not difficult (6). The presence of purpura, along with one of the 4 criteria including abdominal symptoms, joint involvement, kidney damage or the detection of IgA in histological examination, can confirm the diagnosis of IgAV (7). However, in clinical practice, the problem is that for some patients with IgAV, abdominal symptoms occur earlier than the appearance of purpura (8). These symptoms are similar to those of acute abdomen (8), coupled with the poor expressive ability of young children and children, which leads to the possibility of misdiagnosis and delayed diagnosis (9). And the wrong treatment may prolong the patient’s hospital stay, or even cause the disease to worsen. Our previous study reported the characteristics of IgAV patients with abdominal symptoms, but due to the lack of acute abdomen patients as controls, the clinical significance of the study was limited (10).

In this study, children with acute abdominal conditions such as appendicitis and intussusception were included as the control group. The characteristics and diagnostic features of patients with IgAV who present with abdominal symptoms have been described. In addition, a diagnostic model based on clinical symptoms and common laboratory tests has been established, which facilitates the timely diagnosis of IgAV patients, especially those with abdominal symptoms earlier than purpura.

## 2 Methods

### 2.1 Patient

This is a retrospective observational controlled study. All the patients were treated in Nanyang Central Hospital between August 2023 to December 2025. The inclusion criteria include (1): The patient was admitted to our hospital and was first diagnosed with IgAV, appendicitis or intussusception; (2) At the time of the visit, the patient was under the age of 14 and the hospital stay was more than 3 days; (3) All the patients presented with abdominal symptoms, such as abdominal pain, nausea, vomiting and bloody stool; (4) Patients were treated in pediatric departments such as pediatric internal medicine or pediatric surgery in our hospital. The exclusion criteria include: (1) Patients with missing key information in their medical records, such as past medical history and clinical symptoms; (2) Patients with IgAV are also diagnosed with digestive system diseases or other diseases that may cause abdominal symptoms; (3) The patient was diagnosed with other diseases that might affect this study, such as respiratory infections, arthritis, cancer, immune disorders, etc.; (4) The patient had taken medication or received treatment before admission.

The diagnosis of IgAV follows the relevant guidelines in China (11, 12) and is consistent with the Ankara classification standard of 2008 (7). The diagnostic criteria for appendicitis (13) and intussusception (14) are also based on the relevant guidelines. All patients with IgAV who met the above criteria and presented with abdominal symptoms (including those with mixed symptoms) were classified as the IgAV group. Considering the possible differential diagnosis, patients with appendicitis or intussusception who met the above criteria were taken as the control group.

### 2.2 Statement of human rights

This study follows the Declaration of Helsinki and the rights of all patients are protected. Informed consent has been waived. This study was approved by the Ethics Committee of Nanyang Central Hospital (0310142024).

### 2.3 Data collection

The demographic (including age, sex and weight), medical history (including season of onset, clinical symptoms and length of stay) and laboratory tests (including complete blood count, liver and kidney function test and inflammatory indicators) data were collected from medical records. All the laboratory test data were from the patient’s first examination after admission, and the patient had not received any treatment or medication. For patients with IgAV whose abdominal symptoms appeared before the purpura, all the variables were measured before the appearance of purpura. There were missing values in the collected data, mainly including some laboratory test results (for some patients, certain tests were not conducted) and some unclear descriptions of the medical records. The data with more than 30% missing were excluded and the remaining missing data (**Supplementary Table 1**) were interpolated using the R package “missForest” (15) (Version 1.6) and “missForestPredict” (Version 1.0.1) based on the Random Forest method. To avoid data leakage caused by the imputation model, data imputation was completed after the division of the training dataset and the validation dataset. The training dataset was used to establish the imputation model and was applied to interpolate the validation dataset. The outcome variable does not participate in the interpolation throughout the process.

### 2.4 Variable selection and logistic regression analysis

All the data were randomly divided into a training dataset and a validation dataset in 8:2 ratio, and the proportion of the outcome was ensured to be consistent in both datasets. The global randomization seed is set to “666”. The training dataset includes 99 cases of IgAV, 224 cases of intussusception and 188 cases of appendicitis, while the validation dataset contains 24 cases of IgAV, 53 cases of intussusception and 49 cases of appendicitis. After performing missing data imputation on the training dataset separately, Least absolute shrinkage and selection operator (LASSO) regression and Bayesian Information Criterion (BIC) were used for variable selection due to the limited observation incident cases in the training dataset. LASSO regression performed by R package “glmnet” (Version 4.1-10) is used to select variables. In order to retain more variables and enable BIC to perform selection over a wider range, the λ value with the smallest average deviation (lambda.min) was selected, and 21 variables were retained (**Supplementary Figure 1**). Backward Stepwise Regression based on BIC was used to further select the variables. After selecting, 10 variables were finally retained, and the BIC value of the model was 222.5807. Reported regression coefficients, 95% confidence intervals and *P* values are conditional on the selected predictor set and should not be treated as conventional confirmatory inferential statistics, since uncertainty originating from variable screening is not incorporated. All the 10 variables were included in the Multivariate Logistic Regression analysis. Tolerance and variance inflation factor (VIF) were calculated for multicollinearity. The Univariate and Multivariate Logistics Regression model analysis was performed by IBM SPSS statistics 27, and *P* value <0.05 was considered significant.

### 2.5 Establishment of the diagnostic model

A Multivariate Logistic Regression model was established based on the training dataset, and the selected 10 variables were included in the model. For the diagnostic model, continuous variables including Age (year), Time from symptom onset to visit (hour), Monocyte counts (×10^9^/L), Platelet counts (×10^9^/L), Mean platelet volume (fL) and Albumin (g/L) were substituted into the formula as numerical values, and binary variables including Fever, Nausea or vomiting, Hyperactive borborygmi and Hypotonic or absent borborygmi were substituted into the formula with “0” (No) and “1” (Yes). The optimal probability cutoff (0.2055) was determined exclusively on the full 80% training dataset using Youden’s J statistic. The thresholds for all subgroup analyses, 5-fold cross-validation, and validation set analysis are all based on the thresholds of the training dataset model. The predicted probabilities from 5-fold cross-validation were collected and used to evaluate the model along with the predicted probabilities from the validation dataset. Receiver operating characteristic (ROC) curve, calibration curve, decision curve analysis (DCA) (16) and clinical impact curve (CIC) (17) were used to evaluate the performance of the model (18).

### 2.6 Sensitivity and subgroup analysis

To explore the impact of missing value imputation on the model, sensitivity analysis was conducted by filling in the missing values using the minimum, maximum, and median values of the results from each laboratory test respectively. In addition, the complete dataset (with samples containing missing values removed) was also analyzed. The ROC curve and calibration curve were plotted to assess the impact on the model.

To verify the influence of heterogeneity in the control group and age differences on the model, subgroup analyses based on different age groups (the group of patients <= 6 years old and the group of patients > 6 years old) and different control groups (the group of appendicitis patients and the group of intussusception patients) was conducted in training dataset, validation dataset and complete dataset (merge the training set and the validation set). For the subgroup of patients whose abdominal symptoms appeared before the skin symptoms, the analysis was conducted in the complete dataset (all the appendicitis and intussusception patients as control group).

### 2.7 Statistical analysis

Kolmogorov–Smirnov test was used to estimate the distribution of data. For non-normal distributions, Mann–Whitney U test was used to calculate *P* values. For normal distributions, T test was used to calculate *P* values. For count data, Chi-square test and Fisher’s precision probability test were used to calculate *P* values. All the analysis and establishment of model were performed in The R Program Language (Version 4.5.2), R Studio (2026.04.0 Build 526) and IBM SPSS statistics 27. A *P* value < 0.05 was considered significant.

## 3 Results

### 3.1 Characteristics of demographic and clinical symptoms

**Figure 1:**
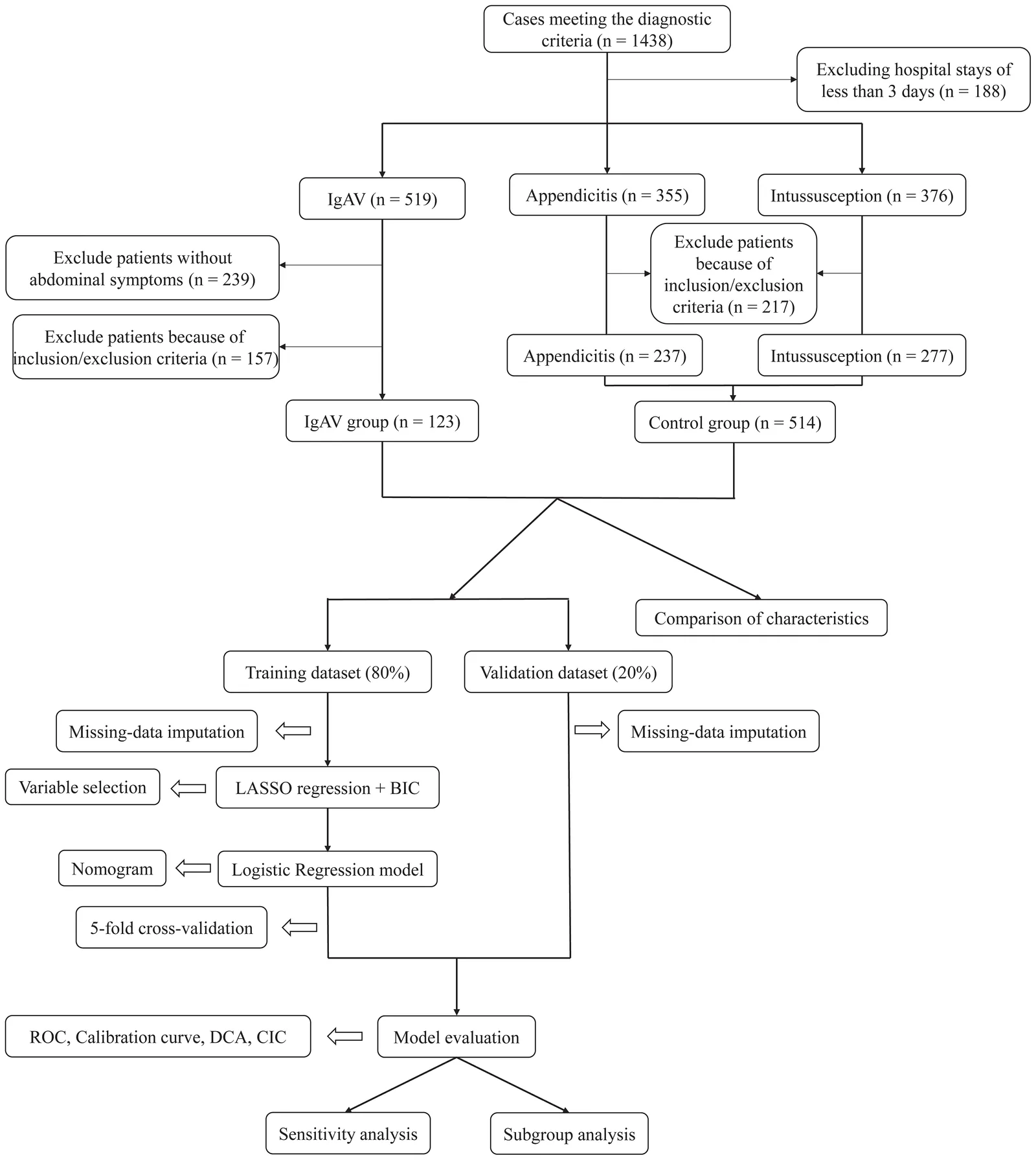
The work flow of case selection, data collection and analysis.

The workflow of this study was shown in the figure (**Figure 1**). Based on the diagnostic and inclusion/exclusion criteria, a total of 637 cases were included in this study, including 123 cases (abdominal symptoms occurred first in 48 cases, purpura occurred first in 31 cases and occurred simultaneously in 44 cases) in the IgAV group and 514 cases (237 cases appendicitis and 277 cases intussusception) in the control group. There were significant differences in demographic information and clinical symptoms between the two groups (**Table 1**). The patients in the IgAV group were over 4 years old at the onset of the disease, while the patients in the control group could potentially develop the disease throughout the infantile period. During the autumn and winter seasons, the incidence rate of IgAV was also higher. The children in the IgAV group seemed to be heavier, but this might be due to the difference in the age of onset.

**Table 1:** Demographic and clinical symptoms data of IgAV group and control group.

Variables	Control group	IgAV group	Z/χ2 value	*P* value
Age (year)	4 (1, 9)	7 (5, 9)	-6.500	< 0.001
Sex			5.281	0.022
female	173 (33.66%)	55 (44.72%)		
male	341 (66.34%)	68 (55.28%)		
Season of onset			18.291	< 0.001
spring	116 (22.57%)	15 (12.2%)		
summer	128 (24.9%)	19 (15.45%)		
autumn	126 (24.51%)	49 (39.84%)		
winter	144 (28.02%)	40 (32.52%)		
Length of stay (day)	6 (5, 9)	7 (6, 9)	-3.318	0.001
Weight (kg)	17.5 (12.0, 30.4)	25.0 (20.0, 32.4)	-6.150	< 0.001
Fever			31.830	< 0.001
no	346 (67.32%)	114 (92.68%)		
yes	168 (32.68%)	9 (7.32%)		
Abdominal pain or crying			0.352	0.553
no	18 (3.5%)	3 (2.44%)		
yes	496 (96.5%)	120 (97.56%)		
Nausea or vomiting			63.768	< 0.001
no	192 (37.35%)	95 (77.24%)		
yes	322 (62.65%)	28 (22.76%)		
Hematochezia			4.221	0.040
no	505 (98.25%)	117 (95.12%)		
yes	9 (1.75%)	6 (4.88%)		
Abdominal distension			0.241	0.623
no	507 (98.64%)	122 (99.19%)		
yes	7 (1.36%)	1 (0.81%)		
Time from symptom onset to visit (hour)	20 (9, 40)	72 (48, 144)	10.416	< 0.001
borborygmi			114.617	< 0.001
normal	208 (40.47%)	110 (95.65%)		
hyperactive	170 (33.07%)	4 (3.48%)		
hypotonic or absent	136 (26.46%)	1 (0.87%)		

Measurement data was shown as median and interquartile range. Mann-Whitney U test and Chi-square test are used to calculate *P* values.

For clinical symptoms, patients in the IgAV group rarely developed fever. Considering that some patients had difficulty expressing their abdominal pain symptoms due to their young age, their crying were also taken into account as manifestations of abdominal pain. Like the control group, most patients in the lgAV group also had abdominal pain symptoms, and a few patients had hematochesia and abdominal distension. Notable differences between the two groups were lower rates of nausea and vomiting and fewer bowel sound changes (including hyperactive, hypotonic or absent) in the IgAV group. The time from symptom onset to visit of patients with IgAV was significantly longer than that of the control group, which may be related to the guardian’s judgment of the severity of symptoms. For patients with abdominal pain, the IgAV group had fewer cases of continuous pain, and the pain location was mainly in the periumbilical (**Table 2**).

**Table 2:** The characteristics and location of patients with abdominal pain in IgAV group and control group.

Variables	Control group	IgAV group	χ2 value	*P* value
Abdominal pain characteristics			84.913	< 0.001
intermittent or episodic	265 (53.43%)	60 (50.00%)		
continuous	176 (35.48%)	8 (6.67%)		
unclear expression or no record	55 (11.09%)	52 (43.33%)		
Location of the pain			132.458	< 0.001
periumbilical	89 (17.94%)	63 (52.50%)		
hypogastrium	218 (43.95%)	7 (5.83%)		
epigastrium	149 (30.04%)	11 (9.17%)		
the entire abdomen	10 (2.02%)	3 (2.50%)		
unfixed or unclear description	55 (11.09%)	39 (32.50%)		

Some patients experience pain in multiple areas simultaneously. Chi-square test and Fisher’s precision probability test are used to calculate *P* values.

### 3.2 Differences in laboratory test data

Compared with the control group, the laboratory test data of the IgAV group showed significant differences (**Table 3**). For the inflammatory-related indicators, the white blood cell count (WBC), neutrophil count (NEU) and monocyte count (MON) were all significantly lower than those in the control group. The lymphocyte count was slightly increased, while CRP did not show any significant change. The red blood cell count, platelet count and mean platelet volume (MPV) did change in IgAV group, but they were all within the normal range. The levels of alkaline phosphatase, albumin and calcium ions were significantly lower in the IgAV group, while the fibrinogen was increased. Notably, IgAV patients had relatively elevated renal function indicators, which may suggest potential renal impairment. In this study, NLR and platelet-to-lymphocyte ratio (PLR) were also calculated, but only the NLR ratio was significantly lower than that of the control group.

**Table 3:** Laboratory test data of IgAV group and control group.

Variables	Control group	IgAV group	t/Z value	*P* value
WBC (×10^9^/L)	11.95 (8.75, 15.51)	10.53 (8.38, 13.35)	-2.468	0.014
NEU (×10^9^/L)	8.64 (5.67, 12.49)	7.27 (5.63, 10.49)	-2.235	0.025
LC (×10^9^/L)	1.85 (1.19, 2.96)	2.18 (1.64, 2.79)	-2.594	0.009
MON (×10^9^/L)	0.60 (0.41, 0.83)	0.52 (0.34, 0.75)	-2.617	0.009
EOS (×10^9^/L)	0.02 (0.00, 0.09)	0.02 (0.00, 0.09)	-0.365	0.715
BAS (×10^9^/L)	0.01 (0.01, 0.02)	0.01 (0.01, 0.03)	-1.852	0.064
RBC (× 10^12^/L)	4.52 (4.22, 4.81)	4.68 (4.39, 4.99)	-3.964	< 0.001
PLT (×10^9^/L)	314 (264, 370)	372 (315, 450)	-6.027	< 0.001
MPV (fL)	8.9 (8.3, 9.6)	9.5 (9.0, 10.4)	-6.297	< 0.001
CRP (mg/L)	5.92 (1.14, 30.05)	7.72 (2.25, 14.86)	-0.372	0.710
ALP (U/L)	205 (166, 242)	180 (141, 226)	-3.677	< 0.001
Albumin (g/L)	45.3 (42.6, 47.6)	41.6 (39.1, 44.9)	-6.381	< 0.001
BUN (mmol/L)	4.07 (3.16, 5.21)	4.10 (3.30, 4.73)	-0.931	0.352
Crea (μmol/L)	28 (22, 37)	36 (31, 44)	-7.317	< 0.001
Ca (mmol/L)	2.47 (2.40, 2.55)	2.38 (2.31, 2.46)	-5.391	< 0.001
FIB (g/L)	3.14 (2.52, 3.98)	3.61 (3.18, 4.17)	-3.763	< 0.001
NLR	4.56 (2.13, 9.35)	3.51 (2.22, 5.59)	-3.005	0.003
PLR	162.58 (103.17, 264.49)	172.13 (123.23, 231.24)	-0.435	0.663

WBC, white blood cell counts; NEU, neutrophil counts; LC, lymphocyte counts; MON, monocyte counts; EOS, eosinophil counts; BAS, basophil counts; RBC, red blood cell; PLT, platelet counts; MPV, mean platelet volume; CRP, C-reactive protein; ALP, alkaline phosphatase; Crea, serum creatinine; BUN, urea nitrogen; Ca, calcium ion concentration; FIB, fibrinogen; NLR, Neutrophil-to-Lymphocyte ratio; PLR, Platelet-to-Lymphocyte ratio. Data was shown as median and interquartile range. Mann–Whitney U test was used to calculate *P* values.

### 3.3 Diagnostic features for IgAV patients with abdominal symptoms

**Figure 2:**
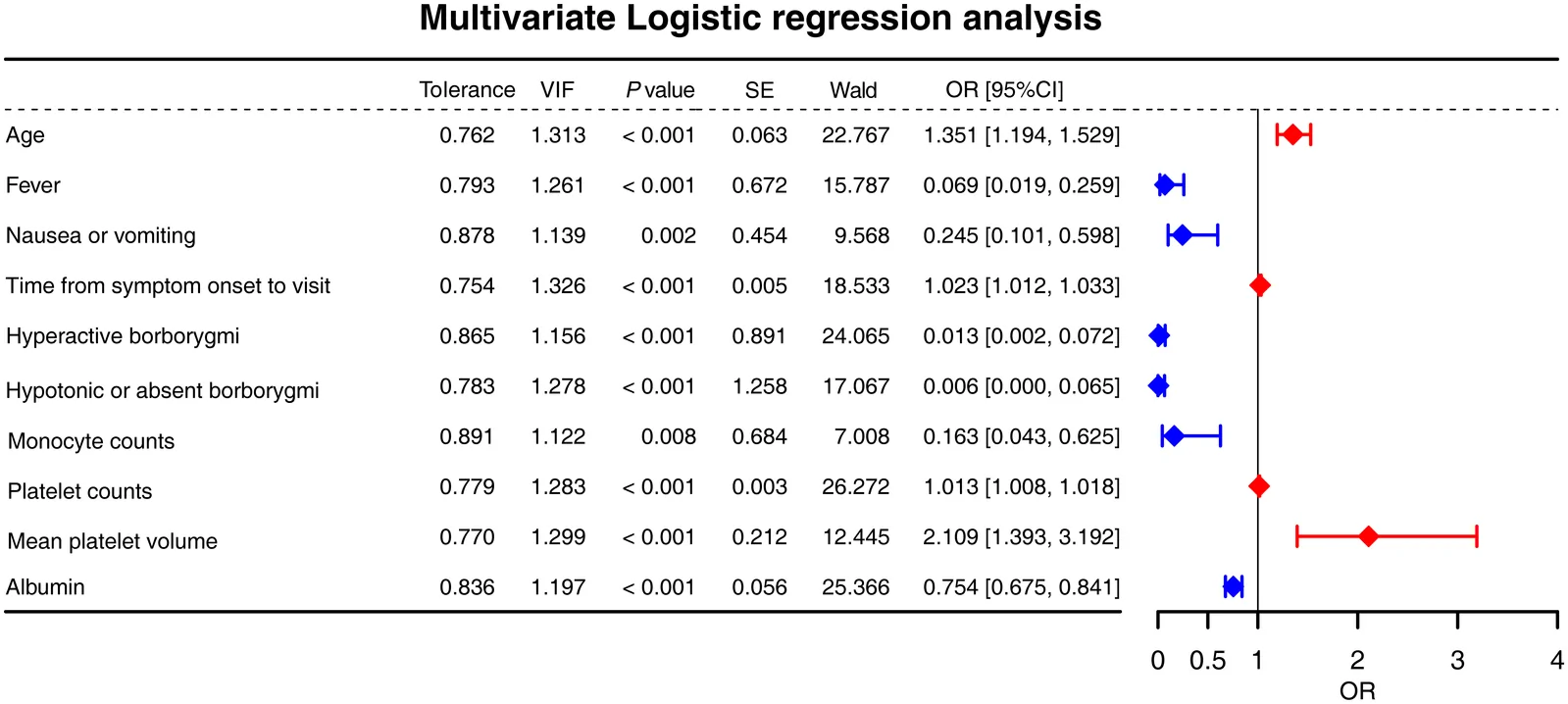
Multivariate logistic regression analysis model of IgAV patients with gastrointestinal symptoms. The red line represents the positively associated with the outcome, and the blue line represents the inversely associated with the outcome.

Based on Univariate Logistics regression analysis, multiple potential diagnostic features were identified (**Supplementary Table 2**). To further determine the independent diagnostic features, LASSO regression and BIC were used to filter variables. Finally, we selected 10 variables without collinearity for Multivariate Logistics regression analysis (**Figure 2**). A total of 4 independent positive correlation diagnostic features and 6 independent negative correlation diagnostic features were identified. Among the 4 independent positive correlation diagnostic features, the older age of the child and the increased MPV had a greater impact on the diagnosis of IgAV. For the 6 independent negative correlation diagnostic features, the occurrence of symptoms such as fever, nausea or vomiting, and changes in borborygmi were related to the diagnosis of appendicitis or intussusception. Elevated albumin and monocyte counts were also more likely to be diagnosed with appendicitis or intussusception.

### 3.4 Diagnostic model for IgAV with abdominal symptoms

**Figure 3:**
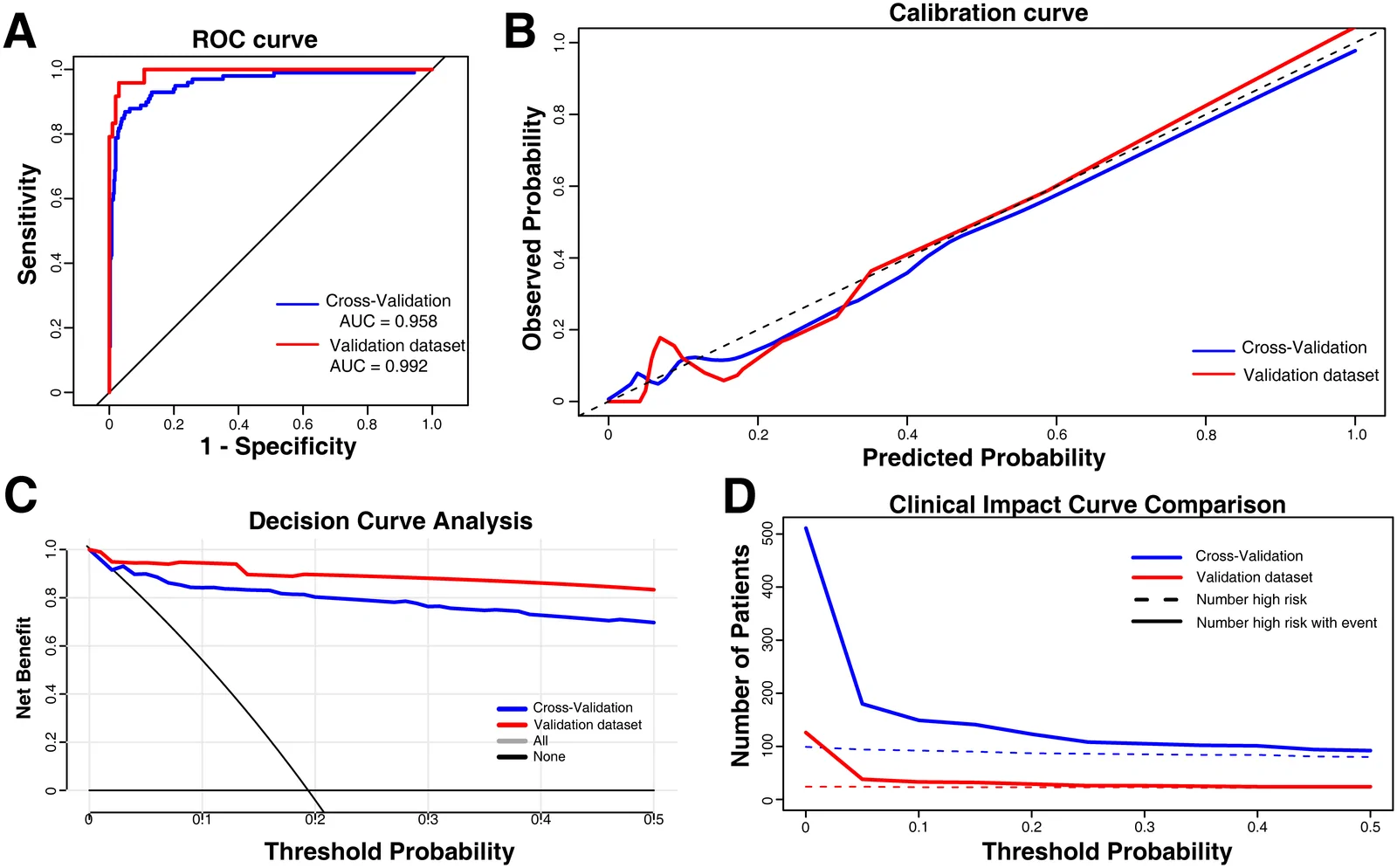
The performance of the multivariate logistic regression model in differential diagnosis IgAV. **(A)** ROC curve analysis of the models in cross-validation and Validation dataset. **(B)** Calibration curve of model in cross-validation and Validation dataset. **(C)** Decision curve analysis of the model in cross-validation and validation dataset. **(D)** Clinical impact curve of the model in cross-validation and validation dataset. ROC, Receiver Operating Characteristic.

**Figure 4:**
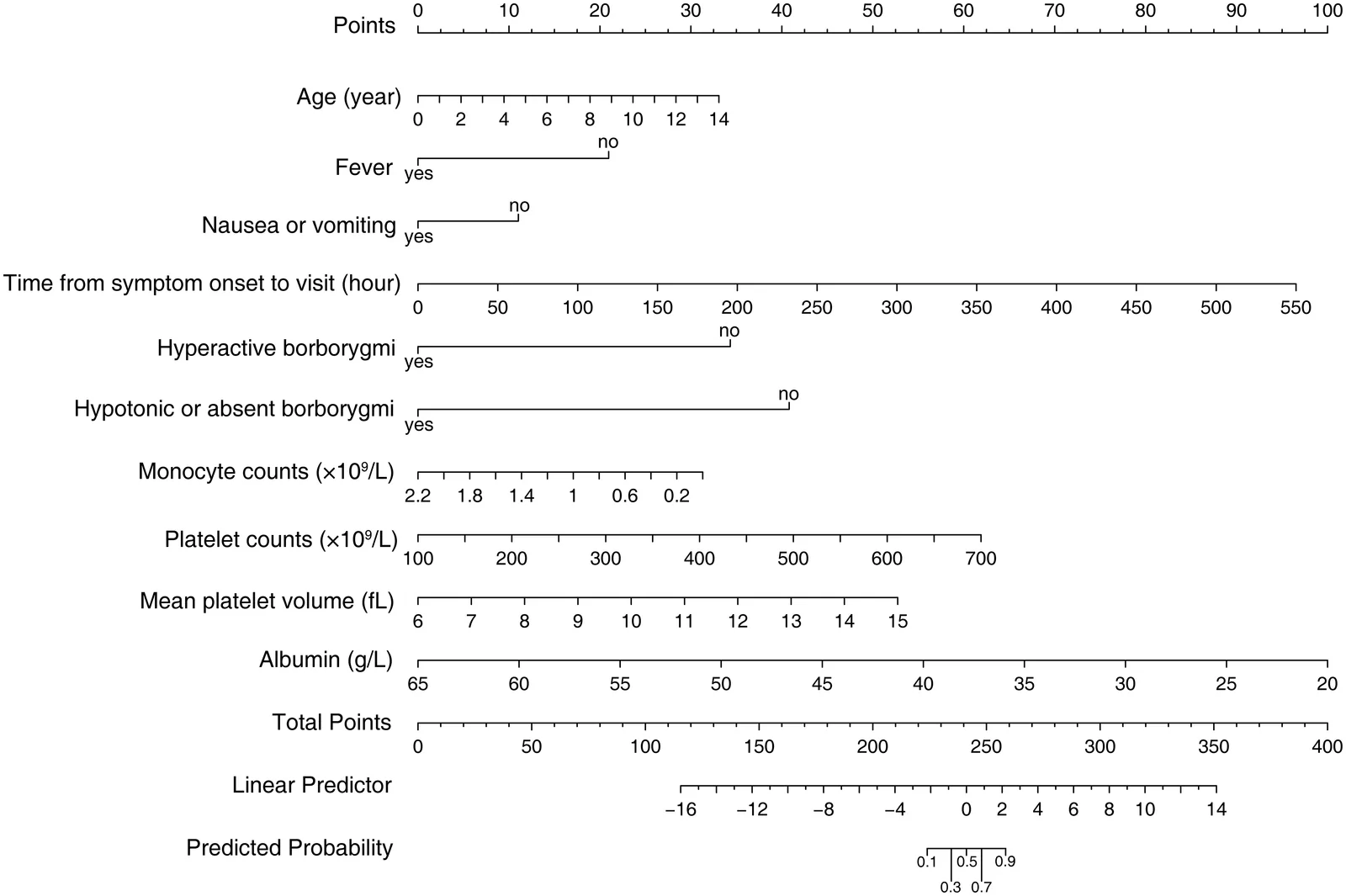
Nomogram of the multivariate logistic regression model.

These 10 features were used to establish a diagnostic model using the training dataset, and the performance of the model was evaluated based on the 5-fold cross-validation method and the validation dataset. The multivariate logistic regression formula was *Logit*(*P*) = –0.4841 + 0.3009×*Age*–2.6686×*Fever*–1.4051×*Nausea or vomiting* + 0.0223×*Time from symptom onset to visit*–4.3712×*Hyperactive borborygmi*–5.1956×*Hypotonic or absent borborygmi*–1.8114×*Monocyte counts* + 0.0131×*Platelet counts* + 0.7461×*Mean platelet volume*–0.2830×*Albumin*. For the ROC analysis, this diagnostic model demonstrated excellent discrimination ability in both the cross-validation (AUC = 0.958, 95%CI: 0.933, 0.983) and validation datasets (AUC = 0.992 95%CI: 0.982, 1.000) (**Figure 3A**; **Table 4**). The model prediction results were also highly consistent with the observed results (**Figure 3B**). The DCA curve analysis indicates that this model was significantly superior to the two control strategies within the entire threshold probability range, both in the cross-validation and in the validation dataset (**Figure 3C**). The CIC analysis showed that the model showed good discrimination ability when the risk threshold exceeded 0.05, which had clinical practical value (**Figure 3D**). Therefore, the nomogram was drawn for convenient application (**Figure 4**). In order to verify the impact of missing value imputation on the model, the minimum, maximum and median imputation and complete dataset were used to conduct sensitivity analysis on the model. Based on the ROC curve and calibration curve analysis, the model still exhibited good performance with different methods of missing value imputation (**Supplementary Figure 2**). Furthermore, the diagnostic performance of NLR was also evaluated (**Supplementary Figure 3**).

**Table 4:** Performance measures of diagnostic model in cross-validation and validation dataset.

Variables	Cross-validation	Validation dataset
AUC (95% CI)	0.958 [0.933, 0.983]	0.992 [0.982-1.000]
Sensitivity	0.879	0.958
Specificity	0.915	0.941
TP	87	23
TN	377	96
FP	35	6
FN	12	1
PPV	0.713	0.793
NPV	0.969	0.99
Calibration intercept	-0.218 [-0.587, 0.161]	0.129 [-1.017, 1.395]
Calibration slope	0.729 [0.593, 0.889]	1.358 [0.800, 2.340]
Brier	0.049	0.025

AUC, Area Under Curve; PPV, Positive Predictive Value; NPV, Negative Predictive Value; TP, True-Positive; TN, True-Negative; FP, False-Positive; FN, False-Negative.

### 3.5 Performance of the diagnostic model in different subgroups

Considering that the onset age of intussusception is often earlier than IgAV, which may lead to an overestimation of the value of age in the diagnostic model, the performance of the model with patients with appendicitis or intussusception as independent controls was verified. The diagnostic model demonstrated excellent performance when used for the separate differential diagnosis of patients with appendicitis and intussusception (**Supplementary Figure 4**; **Supplementary Table 3**). According to the age of the patients at the time of their visit, all patients were divided into two subgroups: those younger than or equal to 6 years old (including 52 cases IgAV and 329 cases control patients) and those older than 6 years old (including 71 cases IgAV and 185 cases control patients). Subgroup analysis based on age stratification also revealed that the model had a good discriminatory performance, whether patients were younger than or equal to 6 years old or older than 6 years old (**Supplementary Figure 5**; **Supplementary Table 4**).

In this study, 48 patients experienced abdominal symptoms before the appearance of purpura, and these were the patients who most required differential diagnosis. For this group of patients, the diagnostic model still demonstrated excellent performance (**Supplementary Figure 6**; **Supplementary Table 5**). Since this subgroup includes some of the samples that were involved in establishing the model, this analysis is an exploratory study rather than an independent validation. Because of the limited sample size, the diagnostic model included all IgAV patients who presented with abdominal symptoms. Therefore, the diagnostic model established is suitable for the differential diagnosis of all IgAV patients with abdominal pain from intussusception and appendicitis. The subgroup analysis for patients who initially presented with abdominal symptoms was exploratory. The model still needs further external validation before widespread clinical application.

## 4 Discussion

Because of the typical and obvious round or oval and erythematous retiform purpura, the diagnosis of IgAV is not difficult (7). The challenge lies in being able to promptly differentiate acute abdominal conditions from the symptoms of purpura in patients whose abdominal symptoms occur before the purpura does. In this study, the demographic data, clinical symptoms and laboratory test data of IgAV patients with abdominal symptoms were systematically compared with those of children with common acute abdominal disorders such as appendicitis and intussusception. IgAV patients with abdominal symptoms usually have an onset age of over 4 years old and are more common in autumn and winter. Compared with patients with acute abdomen, patients with IgAV have lower indicators related to inflammation and a lower proportion of fever, nausea or vomiting, abdominal distension, and changes in borborygmi. Based on these characteristics, 10 related diagnostic features were identified, and a robust diagnostic model was also established, which may be helpful for clinical diagnosis of IgAV patients.

Although abdominal symptoms are common in patients with IgAV, the order in which they and purpura appear varies greatly among studies. In some studies with small sample sizes, only a small number of patients with IgAV initially presented with abdominal symptoms (19, 20). However, in this study, more than one-third of the patients experienced abdominal symptoms as the initial symptom. The differences could be attributed to the variations in sample size, geographical location or different diagnostic criteria. Appendicitis and intussustion are common acute abdominal diseases in children, accounting for about 10% of children’s emergency department visits, and are also the differential diagnosis diseases of IgAV (21). The deposition of lgA immune complexes in the stomach, duodenum and colon may have led to symptoms similar to those of these acute abdominal diseases (2, 22, 23). However, in this study, patients with IgAV had a low proportion of borborygmi changes and nausea or vomiting, and the pain was mainly concentrated in the periumbilical, which can be used as a hint for the differential diagnosis of igAV. Furthermore, indicators related to inflammation, such as WBC, NEU and NLR, showed no significant increase compared to acute abdominal conditions, suggesting that the inflammatory response was not as intense as that in acute abdominal cases. However, patients with IgAV may also further develop complications such as appendicitis and intussusception (24), and whether the conclusions of this study are consistent with patients with complications still needs further study.

There has been concern about IgAV patients with abdominal symptoms, especially those in whom abdominal symptoms first appear (24). Some studies have focused on patients with IgAV combined with gastrointestinal complications (25, 26), but there is still a lack of study on the differential diagnosis between IgAV and acute abdominal diseases. Most studies have the problems of having a small sample size and an inappropriate selection of the control group (26–29). Most studies have focused on the diagnostic value of NLR in patients with IgAV who present with abdominal symptoms (30, 31). However, the clinical value of the NLR was misestimated due to the different selection of the control group. In this study, NLR did not demonstrate good performance in the process of differential diagnosis, which suggests that the application value of NLR in clinical practice is limited. A study used machine learning to establish a differential diagnosis model for IgAV and appendicitis (32), which also shown similar performance (AUC = 0.894, 95%CI: 0.880 - 0.909) to that of our study. Although this study has the advantage of a large sample size, the machine learning method still has the limitation of poor interpretability. Furthermore, the absence of symptoms and characteristics from the model also limits the clinical application value of this study. In contrast, the advantage of this study lies in the establishment of a model and nomogram by integrating clinical symptoms and laboratory test data, which has good medical interpretability and clinical practicability.

In conclusion, this study reveals the differences in the characteristics of IgAV with abdominal symptoms and common acute abdomen in children, which can help in the differential diagnosis. However, in this study, only patients with appendicitis and intussustion were collected as controls, and other acute abdominal diseases such as pancreatitis and intestinal perforation were not collected due to limited numbers, which was a limitation of this study. Furthermore, some inflammatory indicators such as interleukins and PCT were not collected due to insufficient quantities, and future intervention studies may solve this problem. Besides, the limited number of IgAV events may lead to a risk of overfitting in the model, and there is also the limitation of lacking external validation. Since the variable selection and the determination of the probability threshold are both based on the complete training dataset, the estimated results from 5-fold cross-validation may still have a certain degree of selection bias and threshold optimism. The validation dataset is the main basis for evaluating the model’s performance. Overall, this study aims to provide assistance in differential diagnosis of IgAV based on routine physical examinations and laboratory test results.

## Data Availability

The datasets presented in this article are not readily available because All data and R analysis code supporting our findings will be made available upon reasonable request. Requests to access the datasets should be directed to YG (E-mail: Guyuhan1992@outlook.com).
