# Supplementary material for: Clinical characteristics and differential diagnosis model of IgAV with gastrointestinal symptoms and acute abdominal disease: an observational controlled study
Source: Front Immunol. 2026 Aug 28;17:1899915. doi: 10.3389/fimmu.2026.1899915 (PMC13563045; doi:10.3389/fimmu.2026.1899915)
Supplement: Supplementary file 1 [file SupplementaryFile1.docx]

Supplementary Material

1 **Supplementary Figures and Tables**

## 1.1 Supplementary Figures

**Fig. S1 The establishment of Lasso Regression model in diagnosing AIS.** (A) Cross-validation curve. (B) LASSO coefficient path plot.

**Fig. S2 Sensitivity analysis of the Multivariate Logistic regression model.** (A) ROC curve analysis of the model with different missing data handling. (B) Calibration curve of model with different Missing data handling.

**Fig. S3 The performance of the NLR in differential diagnosis IgAV.** (A) ROC curve analysis of the diagnosis with NLR. (B) Calibration curve of diagnosis with NLR.

**Fig. S4 Model performance in subgroup analyses of different control groups.** (A-B) The ROC curve (A) and calibration curve (B) of the diagnostic model in the IgAV and appendicitis subgroups analysis. (C-D) The ROC curve (C) and calibration curve (D) of the diagnostic model in the IgAV and intussusception subgroups analysis.

**Fig. S5 Model performance in subgroup analyses stratified by age.** (A-B) The ROC curve (A) and calibration curve (B) of the diagnostic model in the patients more than 6 years old. (C-D) The ROC curve (C) and calibration curve (D) of the diagnostic model in the patients less than or equal to 6 years old.

**Fig. S6 Model performance in subgroup analyses of patients with abdominal symptoms occurred first.** (A) ROC curve analysis of patients with abdominal symptoms occurred first and Control group. (B) Calibration curve of patients with abdominal symptoms occurred first and Control group.

## 1.2 Supplementary Tables

**Table S1. The number and percentage of missing data.**

|  | **Control group (n = 514)** | **IgAV group (n = 123)** | **Total (n = 637)** |
| --- | --- | --- | --- |
| Age (year) | 0 (0%) | 0 (0%) | 0 (0%) |
| Sex | 0 (0%) | 0 (0%) | 0 (0%) |
| Season of onset | 0 (0%) | 0 (0%) | 0 (0%) |
| Length of stay (day) | 0 (0%) | 0 (0%) | 0 (0%) |
| Weight (kg) | 0 (0%) | 0 (0%) | 0 (0%) |
| Fever | 0 (0%) | 0 (0%) | 0 (0%) |
| Abdominal pain or crying | 0 (0%) | 0 (0%) | 0 (0%) |
| Nausea or vomiting | 0 (0%) | 0 (0%) | 0 (0%) |
| Hematochezia | 0 (0%) | 0 (0%) | 0 (0%) |
| Abdominal distension | 0 (0%) | 0 (0%) | 0 (0%) |
| Time from symptom onset to visit (hour) | 18 (3.5%) | 11 (8.9%) | 29 (4.6%) |
| borborygmi | 0 (0%) | 8 (6.5%) | 8 (1.3%) |
| Abdominal pain characteristics | 18 (3.5%) | 3 (2.4%) | 21 (3.3%) |
| Location of the pain | 29 (5.6%) | 26 (21.1%) | 55 (8.6%) |
| WBC (×10^9^/L) | 4 (0.8%) | 0 (0%) | 4 (0.6%) |
| NEU (×10^9^/L) | 4 (0.8%) | 0 (0%) | 4 (0.6%) |
| LC (×10^9^/L) | 4 (0.8%) | 0 (0%) | 4 (0.6%) |
| MON (×10^9^/L) | 4 (0.8%) | 0 (0%) | 4 (0.6%) |
| EOS (×10^9^/L) | 4 (0.8%) | 0 (0%) | 4 (0.6%) |
| BAS (×10^9^/L) | 4 (0.8%) | 0 (0%) | 4 (0.6%) |
| RBC (× 10^12^/L) | 5 (1%) | 0 (0%) | 5 (0.8%) |
| PLT (×10^9^/L) | 5 (1%) | 0 (0%) | 5 (0.8%) |
| MPV (fL) | 5 (1%) | 0 (0%) | 5 (0.8%) |
| CRP (mg/L) | 8 (1.6%) | 11 (8.9%) | 19 (3%) |
| ALP (U/L) | 14 (2.7%) | 4 (3.3%) | 18 (2.8%) |
| Albumin (g/L) | 57 (11.1%) | 9 (7.3%) | 66 (10.4%) |
| BUN (mmol/L) | 8 (1.6%) | 3 (2.4%) | 11 (1.7%) |
| Crea (μmol/L) | 8 (1.6%) | 3 (2.4%) | 11 (1.7%) |
| Ca (mmol/L) | 66 (12.8%) | 34 (27.6%) | 100 (15.7%) |
| FIB (g/L) | 18 (3.5%) | 38 (30.9%) | 56 (8.8%) |
| NLR | 4 (0.8%) | 0 (0%) | 4 (0.6%) |
| PLR | 5 (1.0%) | 0 (0%) | 5 (0.8%) |

**Table S2. Univariable Logistic regression analysis of the potential risk factors.**

|  | ***P* value** | **SE** | **Wald** | **OR (95%CI)** |
| --- | --- | --- | --- | --- |
| Age | < 0.001 | 0.029 | 36.737 | 1.152 [1.098, 1.230] |
| Sex (male) | 0.088 | 0.228 | 2.919 | 0.678 [0.434, 1.059] |
| Weight | 0.002 | 0.007 | 9.160 | 1.020 [1.007, 1.033] |
| Spring | 0.010 | 0.341 | 6.594 | 0.417 [0.214, 0.813] |
| Summer | 0.023 | 0.302 | 5.193 | 0.503 [0.278, 0.908] |
| Autumn | 0.002 | 0.236 | 9.664 | 2.083 [1.311, 3.308] |
| Winter | 0.144 | 0.239 | 2.139 | 1.418 [0.888, 2.266] |
| Fever | < 0.001 | 0.406 | 22.467 | 0.146 [0.066, 0.324] |
| Abdominal pain or crying | 0.855 | 0.656 | 0.340 | 1.126 [0.317, 3.995] |
| Nausea or vomiting | < 0.001 | 0.259 | 43.193 | 0.183 [0.110, 0.303] |
| Hematochezia | 0.226 | 0.623 | 1.467 | 2.126 [0.627, 7.208] |
| Abdominal distension | 0.624 | 1.075 | 0.240 | 0.590 [0.072, 4.854] |
| Time from symptom onset to visit | < 0.001 | 0.002 | 70.131 | 1.021 [1.016, 1.026] |
| Hyperactive borborygmi | < 0.001 | 0.521 | 20.471 | 0.094 [0.034, 0.263] |
| Hypotonic or absent borborygmi | < 0.001 | 1.011 | 12.235 | 0.029 [0.004, 0.211] |
| WBC | 0.122 | 0.024 | 2.392 | 0.964 [0.921, 1.010] |
| NEU | 0.057 | 0.024 | 3.630 | 0.955 [0.910, 1.001] |
| LC | 0.091 | 0.073 | 2.852 | 1.132 [0.980, 1.307] |
| MON | 0.012 | 0.351 | 6.339 | 0.414 [0.208, 0.822] |
| EOS | 0.757 | 0.008 | 0.096 | 1.002 [0.987, 1.018] |
| BAS | 0.027 | 0.069 | 4.880 | 1.165 [1.017, 1.333] |
| RBC | < 0.001 | 0.260 | 12.352 | 2.498 [1.499, 4.161] |
| PLT | < 0.001 | 0.001 | 31.587 | 1.007 [1.004, 1.009] |
| MPV | < 0.001 | 0.101 | 30.028 | 1.739 [1.427, 2.119] |
| CRP | 0.003 | 0.004 | 8.679 | 0.988 [0.980, 0.996] |
| ALP | 0.016 | 0.002 | 5.789 | 0.995 [0.992, 0.999] |
| Albumin | < 0.001 | 0.026 | 30.350 | 0.868 [0.826, 0.913] |
| Crea | < 0.001 | 0.009 | 11.508 | 1.032 [1.014, 1.052] |
| BUN | 0.548 | 0.074 | 0.360 | 0.956 [0.827, 1.106] |
| Ca | < 0.001 | 0.907 | 17.049 | 0.024 [0.007, 0.140] |
| FIB | 0.057 | 0.089 | 3.624 | 1.185 [0.995, 1.412] |

The Odds Ratio of EOS and BAS were the change amount for every 0.01 increase.

**Table S3. Performance measures of diagnostic model in different Control subgroup.**

|  | **IgAV and Appendicitis** | | | **IgAV and Intussusception** | | |
| --- | --- | --- | --- | --- | --- | --- |
|  | **Train dataset** | **Validation dataset** | **Combined** | **Train dataset** | **Validation dataset** | **Combined** |
| AUC | 0.969 | 0.990 | 0.974 | 0.979 | 0.994 | 0.983 |
| Lower 95% CI | 0.952 | 0.975 | 0.961 | 0.963 | 0.984 | 0.970 |
| Upper 95% CI | 0.986 | 1.000 | 0.988 | 0.995 | 1.000 | 0.995 |
| Sensitivity | 0.929 | 0.958 | 0.935 | 0.929 | 0.958 | 0.935 |
| Specificity | 0.883 | 0.918 | 0.890 | 0.955 | 0.962 | 0.957 |
| TP | 92 | 23 | 115 | 92 | 23 | 115 |
| TN | 166 | 45 | 211 | 214 | 51 | 265 |
| FP | 22 | 4 | 26 | 10 | 2 | 12 |
| FN | 7 | 1 | 8 | 7 | 1 | 8 |
| PPV | 0.807 | 0.852 | 0.816 | 0.902 | 0.920 | 0.906 |
| NPV | 0.960 | 0.978 | 0.963 | 0.968 | 0.981 | 0.971 |
| Calibration intercept | 0.509 | 0.605 | 0.533 | 1.046 | 1.423 | 1.087 |
| Calibration slope | 0.805 | 1.102 | 0.847 | 1.259 | 1.786 | 1.311 |
| Brier | 0.067 | 0.039 | 0.062 | 0.047 | 0.028 | 0.044 |

AUC, Area Under Curve; PPV, Positive Predictive Value; NPV, Negative Predictive Value; TP, True‑Positive; TN, True‑Negative; FP, False‑Positive; FN, False‑Negative.

**Table S4. Performance measures of diagnostic model in different age subgroup.**

|  | **≤6 years old** | | | **>6 years old** | | |
| --- | --- | --- | --- | --- | --- | --- |
|  | **Train dataset** | **Validation dataset** | **Combined** | **Train dataset** | **Validation dataset** | **Combined** |
| AUC | 0.979 | 0.998 | 0.982 | 0.964 | 0.994 | 0.972 |
| Lower 95% CI | 0.959 | 0.992 | 0.966 | 0.940 | 0.980 | 0.953 |
| Upper 95% CI | 0.999 | 1.000 | 0.999 | 0.989 | 1.000 | 0.990 |
| Sensitivity | 0.909 | 1.000 | 0.923 | 0.945 | 0.938 | 0.944 |
| Specificity | 0.970 | 0.952 | 0.967 | 0.834 | 0.925 | 0.854 |
| TP | 40 | 8 | 48 | 52 | 15 | 67 |
| TN | 259 | 59 | 318 | 151 | 37 | 158 |
| FP | 8 | 3 | 11 | 24 | 3 | 27 |
| FN | 4 | 0 | 4 | 3 | 1 | 4 |
| PPV | 0.833 | 0.727 | 0.814 | 0.684 | 0.833 | 0.713 |
| NPV | 0.985 | 1.000 | 0.988 | 0.976 | 0.974 | 0.975 |
| Calibration intercept | 0.555 | 2.898 | 0.624 | -0.201 | 0.018 | -0.154 |
| Calibration slope | 1.383 | 4.584 | 1.491 | 0.781 | 0.988 | 0.819 |
| Brier | 0.028 | 0.018 | 0.026 | 0.063 | 0.034 | 0.057 |

AUC, Area Under Curve; PPV, Positive Predictive Value; NPV, Negative Predictive Value; TP, True‑Positive; TN, True‑Negative; FP, False‑Positive; FN, False‑Negative.

**Table S5. Performance measures of diagnostic model in patients with abdominal symptoms occurred first and Control group.**

|  | Abdominal symptoms occurred first |
| --- | --- |
| AUC (95%CI) | 0.991 (0.983, 0.998) |
| Sensitivity | 0.979 |
| Specificity | 0.926 |
| TP | 47 |
| TN | 476 |
| FP | 38 |
| FN | 1 |
| PPV | 0.553 |
| NPV | 0.998 |
| Calibration intercept | -1.149 |
| Calibration slope | 1.210 |
| Brier | 0.023 |

AUC, Area Under Curve; PPV, Positive Predictive Value; NPV, Negative Predictive Value; TP, True‑Positive; TN, True‑Negative; FP, False‑Positive; FN, False‑Negative.
